# Enhancement of Immune Activities of Mixtures with *Sasa quelpaertensis* Nakai and *Ficus erecta* var. *sieboldii*

**DOI:** 10.3390/foods9070868

**Published:** 2020-07-02

**Authors:** Hee-Yeon Kwon, Sun-Il Choi, Xionggao Han, Xiao Men, Gill-Woong Jang, Ye-Eun Choi, Seung-Hyung Kim, Jun-Chul Kang, Ju-Hyun Cho, Ok-Hwan Lee

**Affiliations:** 1 Department of Food Science and Biotechnology, Kangwon National University, Chuncheon 24341, Korea; sosakwon1@naver.com (H.-Y.K.); docgotack89@hanmail.net (S.-I.C.); xionggao414@hotmail.com (X.H.); menxiaodonglei@naver.com (X.M.); jkw5235@naver.com (G.-W.J.); ye0538@daum.net (Y.-E.C.); 2 Institute of Traditional Medicine and Bioscience, Daejeon University, Daejeon 34520, Korea; sksh518@dju.kr; 3 Haram Central Research Institute, Cheongju 28160, Korea; Kang.jc@eharam.com

**Keywords:** immune activities, *Sasa quelpaertensis* Nakai, *Ficus erecta* var. *sieboldii*, RAW264.7, mixture

## Abstract

The objective of the present study was to develop a concoction of natural products that could dramatically improve immune function with minimal possible side effects. *Sasa quelpaertensis* Nakai and *Ficus erecta* var. *sieboldii* are plants that are native to Jeju Island, Korea and are known to be rich in physiologically active substances. We prepared a mixture of different proportions and extraction conditions using two natural plants and determined their optimum mixing ratio and extraction method by assessing immune function-related biomarkers in RAW264.7 macrophages. Optimal extract (HR02/04(8:2)-W) was selected from in vitro experiments and its immunity-enhancing efficacy was evaluated in mice. After oral administration of extract to BALB/c mice for 2 weeks, nitric oxide production in the peritoneal exudate cells, natural killer cell cytotoxicity, cytokine expression in splenocytes, and total cell number of immune tissues and phenotype analysis were evaluated. Our results demonstrated that HR02/04(8:2)-W significantly enhanced the immune system by increasing natural killer cell activity, cytokine expression, and total number of cells in immune tissues. In conclusion, our study validates the role of HR02/04(8:2)-W in enhancing immunity and its potential development as a functional food.

## 1. Introduction

Immunity plays a key role in the host defense system against antigens such as microorganisms and other substances that either result from the invasion of hostile metabolites or on the generation of exogenous infectious substances within the host. The immune response is divided into two categories: innate immunity and adaptive immunity [1,2]. Innate immunity primarily involves the presence of macrophages or natural killer (NK) cells and predominantly combats non-specific pathogens. Adaptive immune response mainly involves immune T cells (cell-mediated) and immune B cells (body fluid-mediated). Furthermore, T cells have been largely divided into helper T (Th) cells and cytotoxic T (Tc) cells [3]. Th cells have been known to mediate cellular as well as fluid immune responses and are further categorized into Th1 and Th2 cells based on cytokine secretion. Th1 cells have been shown to express interleukin-2 (IL-2), interferon-γ (IFN-γ), and tumor necrosis factor (TNF), which stimulate phagocytosis in lymphocytes. Moreover, Th2 cells have been reported to have the ability to promote B-cell activities including production of antibodies by secreting interleukin-4 (IL-4) and interleukin-10 (IL-10) [3]. Thus, in the immune system, there is a direct interaction between different cytokines and cells and thereby functions to protect host systems from pathogens.

Recently, modern society has been exposed to an environment that can easily weaken biological immune functions due to aging, environmental pollutants, stress, and medicinal side effects. Thus, increasing scientific interest has been observed in foods and drugs that can improve the immune system [4]. As a result, research has been actively conducted to enhance immunity or restore degraded immune functions by using immune boosters that are derived from natural substances with minimal possible side effects [5,6,7].

*Ficus erecta* var. *sieboldii* (FES) is a plant that is native to Jeju Island and the southern coastal regions, especially Jeju Island in Korea. For a long time, it has been known to be effective in promoting rejuvenation, bone health, wound recovery, blood circulation, and detoxification [8]. In recent studies, the potential of FES as a functional food was reported by evaluating its catechin content, antioxidant activity, and anti-inflammatory effects in a hypersensitivity-free macrophage model [9,10]. *Sasamorpha* has been reported to be widely distributed in Asian countries such as Korea, China, and Japan [11]. Since ancient times, fruits have been shaped like barley or wheat grains and contain starch. Thus, these fruits have been reported to be used earlier as food products. Furthermore, leaves have been used as exclusive medicine for treating inflammation, fever, and diuretic effects [12]. Several health promoting effects have been reported using cell model and animal model experiments, which include antioxidant, antibacterial, anti-inflammatory, and anti-obesity activities [12,13,14,15]. Among the species, *Sasa quelpaertensis* Nakai (SQN) is a special plant that is native to Mt. Halla on Jeju Island. Recently, extracts of its leaves have been reported to have various physiological activities such as anti-obesity, antioxidant, anti-inflammatory, and anti-cancer. Moreover, they have been used as health-enhancing food and cosmetic materials [16,17,18,19].

It has been widely claimed that when food ingredients are mixed rather than using a single material, the synergistic therapeutic effects due to the combination of biochemicals may be superior to that of single biochemicals [20,21]. Much research has reported that when the mixed material is treated, a more effective bioactive effect is exhibited than when a single material is treated [22,23,24]. In this study, we aimed to establish a mixing ratio that dramatically enhanced immune function by utilizing two natural resources of Jeju, FES and SQN. Additionally, using in vitro and in vivo experiments, we showed its remarkable potential as a functional food and being developed into materials that could help improve immunity.

## 2. Materials and Methods 

### 2.1. Preparation of Samples 

FES and SQN were obtained from SamWon Nature (Jeju, Korea). FES and SQN were blended in distilled water (1:20) with different mixing ratios (10:0, 2:8, 5:5, 8:2, and 0:10). Further, hot water extract was prepared by extracting the mixture in a reflux extraction device at 95 °C for 4 h. To prepare an ethanol extract, 70% ethanol was added to the mixture (1:20) and extracted at 85 °C in a reflux extraction device for 4 h. Each method was performed twice and the extracts that were obtained in the first and second stages were mixed, filtered using a filter paper (ADVANTEC, Tokyo, Japan), and then concentrated to a specific concentration (Eyela, Tokyo, Japan). Further, they were dried using a freeze dryer (Labconco, Kansas, MO, USA) and eventually, hot water extract powder samples (HR1902-W, HR02/04(2:8)-W, HR02/04(5:5)-W, HR02/04(8:2)-W, HR1904-W) and 70% ethanol extract powder samples (HR1902-70E, HR02/04(2:8)-70E, HR02/04(5:5)-70E, HR02/04(8:2)-70E, HR1904-70E) were obtained. Yield for each extract was calculated as a percentage of the original weight of the extract (Table 1). Korea red ginseng, which was used as a positive control, was purchased at Geumsan Red Ginseng Land (Geumsan, Korea) and was diluted and treated to appropriate concentrations for cell treatment and animal administration. Rutin, an active component of the FES and SQN, was purchased from Sigma-Aldrich (St. Louis, MO, USA) and was diluted to appropriate concentrations for mouse administration.

### 2.2. Cell Culture

RAW264.7, a murine macrophage cell line, was purchased from Korea Cell Line Bank (KCKB, Seoul, Korea) and cultured in the Dulbecco’s Modified Eagle Medium (Hyclone Laboratories, Logan, UT, USA) containing 10% fetal bovine serum (Gibco RBL Co., Grand Island, NY, USA) and 100 units/mL penicillin-streptomycin (Hyclone Laboratories, Logan, UT, USA) at 37 °C in a 5% CO_2_ incubator (311GP, Thermo Fisher Scientific, Waltham, MA, USA).

### 2.3. Cell Viability

Cells were plated in a 96-well plate (Corning Inc, Cambridge, MA, USA) at a density of 5 × 10^4^ cells/mL and incubated for 24 h. Further, cells were treated with each sample for 24 h at a concentration of 10 μg/mL, 100 μg/mL, and 1000 μg/mL. MTT assay kit (Dozen, Seoul, Korea) was used to measure cytotoxicity at an absorbance of 450 nm using a microplate reader (Epoch, Biotek Instruments Inc., Winooski, VT, USA) [25].

### 2.4. Measurement of Nitric Oxide (NO) Production In Vitro

Cells were plated in a 48-well plate at a density of 5 × 10^4^ cells/mL and incubated for 24 h. Further, cells were treated with each sample for 48 h at a concentration of 10 μg/mL, 100 μg/mL, and 1000 μg/mL. Subsequently, 100 μL of the supernatant was transferred to a 96-well plate and assessed using a NO assay kit (Intron, Sungnam, Korea). Absorbance was measured at 540 nm using a microplate reader [26].

### 2.5. Enzyme-Linked Immunosorbent Assay (ELISA) on RAW 264.7 Cells

Cells were plated in a 48-well plate at a density of 5 × 10^4^ cells/mL and incubated for 24 h. Further, each sample was treated for 48 h at a concentration of 10 μg/mL, 100 μg/mL, and 1000 μg/mL. Subsequently, 100 μL of supernatant was transferred to a 96-well plate and interleukin-1β (IL-1β), interleukin-6 (IL-6), and TNF-α expression levels were measured using an ELISA kit (R&D systems, Minneapolis, MN, USA) [27].

### 2.6. Animal Experiments

Eight-week-old male BALB/c mice were purchased from Daehan Bio Link (Eumseong, Korea). Water and solid feed were allowed for consumption without restriction. Animals were nurtured in a laboratory environment at a temperature of 23 °C ± 2 °C and humidity of 50% ± 10% under a 12 h light/12 h dark cycle. They were fed with a standard laboratory diet and provided with water ad libitum. Before proceeding with the experiments, all animals were quarantined for at least 7 days for environmental adaptation and stabilization of health. This experiment was conducted in accordance with the regulations set by the Animal Experimental Ethics Committee of Kangwon National University (approval number: KW-191119-2). All mice were sacrificed using CO_2_ inhalation.

After one week of adaptation, all mice were randomly divided into seven groups (*n* = 5/group). Group 1 mice were administered with a normal diet ad libitum as a control. Group 2 and 3 mice were administered with a normal diet ad libitum with 100 mg/kg and 200 mg/kg KRG, respectively. Group 4, 5, and 6 mice were administered with a normal diet ad libitum with 50 mg/kg, 100 mg/kg, and 200 mg/kg HR02/04(8:2)-W, respectively. Group 7 mice were administered with a normal diet ad libitum with 10 mg/kg rutin. All mice received samples by oral gavage for 2 weeks. On the day after the oral administration course was completed, tissues were excised from mice corresponding to each experiment.

### 2.7. Measurement of NK Cell Cytotoxicity

NK cell activity of splenocytes (effector cells) was analyzed using YAC-1 cells (target cells) [28]. Splenocyte suspensions were co-cultured with YAC-1 cells to obtain an effector-to-target cell ratio of 50:1 in 96-well culture plates in a CO_2_ incubator (37 °C, 5% CO_2_) for 4 h. Further, supernatant was mixed with lactate dehydrogenase (LDH) solution. Absorbance was measured at 490 nm and the percentage of NK cell activity was calculated using the following formula: NK cell cytotoxicity (%) = [(experimental release-spontaneous release)/(maximum release-spontaneous release)] × 100.

### 2.8. ELISA on Spleen Cells 

Expression of IL-2, IL-4, IL-10, IL-12, and IFN-γ was evaluated using spleen cells, which were suspended in RPMI 1640 medium containing 2 mM L-glutamine and 5% fetal bovine serum. Further, spleen cells were cultured for 48 h at a density of 1 × 10^5^ cells/well in a 96-well culture plate. After 48 h, supernatant was collected and assessed using an ELISA kit.

### 2.9. Measurement of NO Production In Vivo 

Three days before the end of the experiment (day 11), 1.5 mL of 10% aqueous solution of proteose peptone (Difco Laboratories Inc., Detroit, MI, USA) was administered into the intraperitoneal cavity of mice and the abdomen was then sterilized with alcohol. After exposing the peritoneum, 4.5 mL of cooled Hanks’ balanced salt solution (HBSS, Sigma, St. Louis, MO, USA) was injected into the abdominal cavity with a syringe. The abdomen was massaged for 30 s and then fluid was recovered using a syringe. The recovered cells were washed twice with HBSS solution, suspended in the RPMI 1640 medium, and seeded (200 µL) in a 96-well culture dish at a final concentration of 2 × 10^6^ cells/well. Suspended cells were incubated for 2 h in a CO_2_ incubator (37 °C, 5% CO_2_) for attachment of macrophages. Subsequently, suspended cells were removed by washing three times with HBSS solution and 100 μL of the RPMI 1640 medium was added to each well. After 72 h of incubation, 100 μL of the culture supernatant was collected and incubated with the Griess reagent (0.1% NEDHC [*n*-1-(naphthyl)-ethylene diamide and sulfanilamide in 5% H_3_PO_4_]) for 10 min at room temperature. Subsequently, the colorimetric reaction was measured at 540 nm.

### 2.10. Immune Cell Phenotype Analysis

Two days before the end of the experiment (day 12), 0.5 mL of 2% aqueous solution of starch was administered into the intraperitoneal cavity of mice. Peyer’s patches, spleen, thymus, draining lymph node (DLN), and peritoneal exudate cell (PEC) were excised from mice, constrained through a 100-μm nylon mesh cell strainer in chilled HBSS and filtered through cotton wool in a syringe barrel. Further, cells were washed and resuspended in the ACK Lysis Buffer (Thermo Fisher Scientific, Waltham, MA, USA) for 5 min and then immediately washed twice in HBSS. Freshly isolated murine cells (5 × 10^5^ cells) were resuspended in 100 μL of the FACS Stain Buffer (BD Biosciences, San Jose, CA, USA) per assay tube [29]. Isolated cells were washed twice with the FACS buffer and then immunofluorescence staining was performed at 4 °C. After reacting with primary antibodies for 30 min, cells were washed with PBS three or more times and then measured with FACSCalibur^®^ (BD Bioscience, San Jose, MA, USA). Percentage of CD3e+/CD19+, CD3e+/CD49b+, CD4+/CD8+, CD4+/CD25+, CD8+/CD25+, CD23+/B220+, CD69+/B220+, CD69+/CD11b+, Gr-1+/CD11b+, and CD4+/CD62L+/CD107a+ cells was analyzed using the Cell Quest program, and the wavelength expressed in each cell was represented by a dot plot. All antibodies for flow cytometric analysis were purchased from Becton Dickinson PharMingen (San Diego, CA, USA). Absolute cell numbers were counted manually using a hemocytometer chamber (Thermo Fisher Scientific, Waltham, MA, USA). Cells (2 × 10^3^) were then centrifuged to attach them onto glass slides (Cellspin, Hanil, Yangju, Korea) (400× *g* for 4 min). Differential count was prepared according to standard morphological criteria. Absolute numbers of various immune cells were quantified by multiplying total number by gating percent (CD area in FACS data).

### 2.11. Statistical Analysis

For data analysis, *p*-values were analyzed using unpaired Student’s *t*-test software (Startview 5.1; Abacus Concepts, Berkeley, CA, USA). Results were considered statistically significant if *p* < 0.05 (*), <0.01 (**), or <0.001 (***).

## 3. Results and Discussion

### 3.1. Effect of Mixtures on RAW264.7 Cells Viability

MTT assay was performed to evaluate the effect of mixtures on the viability of RAW264.7 cells. Results showed that mixtures concentrated in the range of 1000 μg/mL did not affect the viability of RAW264.7 macrophages (data not shown). Based on the data, concentrations of 10 μg/mL, 100 μg/mL, and 1000 μg/mL were selected for subsequent experiments.

### 3.2. Effect of Mixtures on NO Production of RAW264.7 Cells

**Figure 1:**
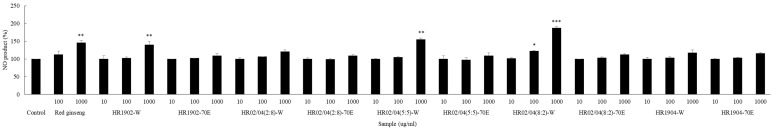
Effect of mixtures on the NO production by RAW264.7 macrophage. The NO production of RAW264.7 cells incubated with each sample for 48 h was measured using an NO assay kit. Data are shown as mean ± SD. * *p* < 0.05, ** *p* < 0.01, and *** *p* < 0.001 compared with the normal control group.

NO production is a feature of immune cells (NK cells, mast cells, and phagocytic cells). NO is a highly reactive molecule that is synthesized by nitric oxide synthase (NOS). It promotes relaxation of blood vessels, neurotransmission, and immune responses between cells. Surplus NO is produced by inducible NOS (iNOS), specifically when microbial pathogens penetrate the body. Subsequently, this abundance of NO expression has been reported to serve as a crucial mediator while attacking pathogens such as bacteria, viruses, fungi, and parasites [30]. Figure 1 shows data representing the capability of RAW264.7 cells to produce excess NO. Results confirmed that the experimental groups treated with HR1902-W (1000 μg/mL), HR02/04(5:5)-W (1000 μg/mL), and HR02/04(8:2)-W (100 and 1000 μg/mL) had significantly upregulated NO production compared to that in the control group. Among them, the HR02/04(8:2)-W (1000 μg/mL) treatment group was found to have significant (>88%) upregulation in NO production compared to that in the control group, which was observed to be the highest increase among that observed in all the experimental groups. When the HR1902 and HR1904 were treated alone, the maximum was measured at 140% and 118%, respectively. Compared to this, the mixture effectively stimulated NO production up to 40% or more than a single extract and showed a strong synergistic effect. Moreover, compared to the 146% increase in NO production of the 1000 μg/mL KRG group (positive control) over the control group, the HR02/04(8:2)-W-treated group was found to have a 40% additional increase in NO production than the KRG-treated group. Thus, results suggested that the mixing ratio was remarkably effective in boosting the immune system of HR02/04(8:2)-W-treated cells.

### 3.3. Effect of Mixtures on Cytokines Production of RAW264.7 Cells

To investigate whether mixtures influenced the release of cytokines in RAW264.7 cells, we assessed IL-1β, IL-6, and TNF-α expression using ELISA kits. IL-1β is a member of the interleukin 1 family cytokine, which is produced by macrophages, neutrophils, and epithelial cells. IL-1β has been reported to be a vital mediator of the inflammatory response and is also known to be involved in various cellular activities such as cell proliferation, differentiation, and apoptosis [31]. Furthermore, IL-6 is a cytokine that is mainly produced by macrophages and T lymphocytes and contributes towards innate immunity as well as adaptive immunity. During innate immune response, IL-6 induces the production of neutrophils from bone marrow precursor cells. In adaptive immunity, IL-6 has been shown to stimulate the maturation of B lymphocyte and enhance their capability to produce antibodies. It has also been reported to promote cellular immunological reactions by inhibiting the synthesis and function of regulated T lymphocytes [32]. TNF-α is a pleiotropic polypeptide that plays a significant role in immune system and inflammatory activities. TNF-α is mainly secreted by activated macrophages, but is also produced by a variety of cells such as Th cells and NK cells. TNF-α has been shown to have the ability to induce pathogenic death, inhibit tumorigenesis, or repress viral replication through production of IL-1 and IL-6 [33].

**Figure 2:**
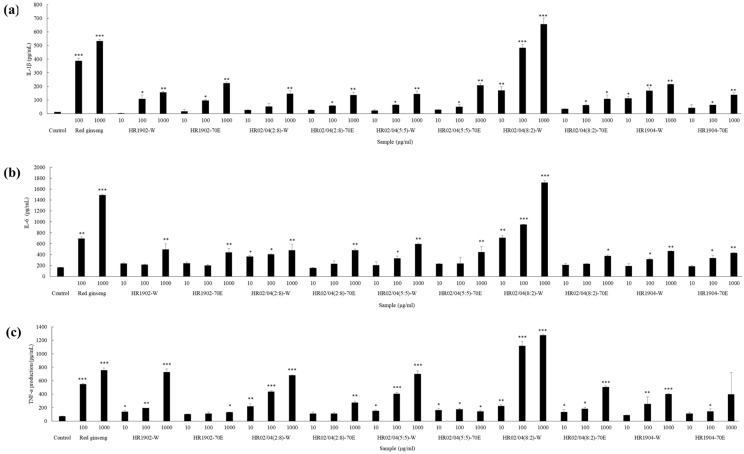
Effect of mixtures on IL-1β (**a**), IL-6 (**b**), and TNF-α (**c**) production by RAW264.7 macrophage. Production of IL-1β, IL-6, and TNF-α were measured in the medium of RAW264.7 cells cultured with each sample for 48 h. Data are shown as mean ± SD. * *p* < 0.05, ** *p* < 0.01, and *** *p* < 0.001 compared with the normal control group.

Data indicating the expression of IL-1β, IL-6, and TNF-α, which are the key factors of the immune response, are shown in Figure 2. Results confirmed that IL-1β expression levels (Figure 2a) were significantly upregulated in all experimental groups compared to the control group. When HR1902 and HR1904 were treated alone, the maximum was measured at 223.4 pg/mL and 215.6 pg/mL, respectively. However, the mixed extract of the two showed a synergetic effect, which increased the expression of IL-1β very effectively up to 655.8 pg/mL in HR02/04(8:2)-W. Moreover, it was found that IL-1β expression levels were significantly more upregulated in HR02/04(8:2)-W than the KRG-treated group (about 97.1 pg/mL at 100 μg/mL and approximately 122.3 pg/mL at 1000 μg/mL). When cells were treated with samples at 1000 μg/mL, the IL-6 expression level was observed to be significantly increased compared to the control group (Figure 2b). When HR1902 and HR1904 were treated alone, they were measured to be 497.2 pg/mL and 465.3 pg/mL, respectively. However, the mixed extract of the two was measured at a maximum of 1721.3 pg/mL, showing a strong synergetic effect, and increased the expression level of IL-6 by three times or more than a single extract. Additionally, it showed an increase of about 231.5 pg/mL compared to that observed in the KRG-treated group and effectively enhanced the expression level of IL-6 in macrophages. Further analysis showed that when cells were treated with HR1902-W, HR02/04(2:8)-W, and HR02/04(5:5)-W (each at 1000 μg/mL), TNF-α expression levels were found to be 726.9 pg/mL, 681.4 pg/mL, and 702.2 pg/mL, respectively, which was similar to that observed in the KRG-treated group (757.1 pg/mL). Experimental groups that were treated with HR02/04(8:2)-W extracts at concentrations of 100 μg/mL and 1000 μg/mL were found to have more than 1.5-fold upregulation in the TNF-α expression level than those in the KRG-treated group. Thereby, this mixing ratio was found to effectively improve the immune response in macrophages. Furthermore, experiments showed that hot water extracts tended to stimulate immune-related biomarkers more effectively than ethanol extracts. Consistent with the highest NO expression exhibited by HR02/04(8:2)-W-treated cells, cytokine expressions were also found to have the most significant upregulation on HR02/04(8:2)-W treatment. Consequently, hot water extract (8:2) of HR1902 and HR1904 was considered to have the most immune-enhancing efficacy and thus, was used for further in vivo experiments. 

### 3.4. NK Cytotoxicity Activity

**Figure 3:**
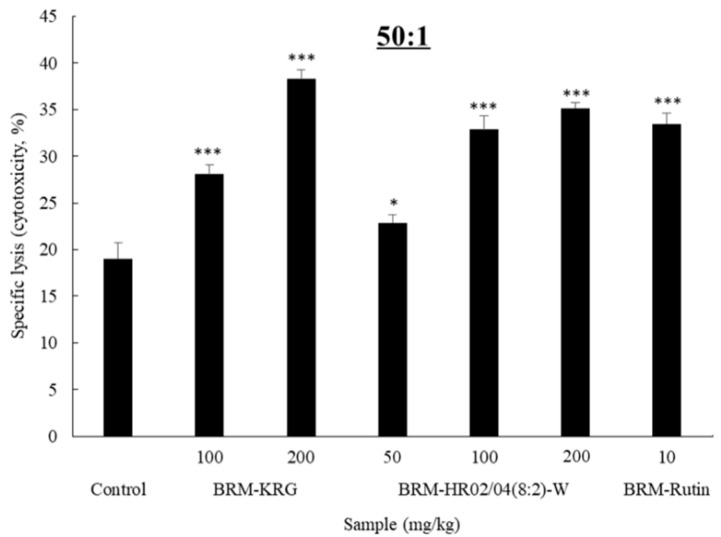
Effect of HR02/04(8:2)-W on enhanced NK cell cytotoxicity. After oral administration for 2 weeks, the splenocytes were co-cultured with target cells (YAC-1) in 96-well plates for 4 h with a ratio of effector to target cells of 50:1. Data are shown as mean ± SD. * *p* < 0.05, *** *p* < 0.001 compared with the normal control group.

NK cells are known to have remarkable abilities to eliminate cancer cells. They comprise 5–10% of the lymphocytes in the blood and are reported to have natural immune function that directly destroys viruses or infected cells without prior detection of specific antigens [34]. Moreover, since NK cells are lymphocytes that secrete cytokines and also contribute to adaptive immunity by stimulating Tc cell response, they have been observed to play an important role in the immune response mechanisms [35]. Data indicating the cytotoxicity capability of NK cells is shown in Figure 3. When mice were administered with HR02/04(8:2)-W at 50 mg/kg, 100 mg/kg, and 200 mg/kg, NK cell-induced cytotoxicity was found to be 22.87%, 32.82%, and 35.15%, respectively. On comparing the cytotoxicity in the experimental group with 19.01% cytotoxicity in the control group, it was confirmed that the probability of apoptosis was significantly increased in the HR02/04(8:2)-W-treated group. The KRG-treated group was used as a positive control, which was found to be 28.07% and 38.26% cytotoxic at concentrations of 100 mg/kg and 200 mg/kg, respectively. Thus, HR02/04(8:2)-W was considered to have the ability to enhance immunity by increasing the activity of NK cells to a level similar to that of the KRG-treated group. Furthermore, on treatment with rutin, which is a biologically active component of the HR02/04, we found high NK cell cytotoxicity. As a result of the experiment, it was confirmed that HR02/04(8:2)-W effectively increased NK cytotoxicity and the superior immune-enhancing effect of HR02/04(8:2)-W might be due to the interaction between rutin and complex components [9].

### 3.5. Effect of HR02/04(8:2)-W on the Expression of Cytokines in Splenocytes

Cell–cell communication within the immune system is mediated by various factors called cytokines. These molecules exert their biological functions through specific receptors, which are expressed on the surface of the target cells. Interaction of these cytokines with each other is complex and has been shown to play an important role in the regulation of immune function by controlling the differentiation and growth of lymphocytes [36]. To investigate whether HR02/04(8:2)-W was related to T cell activity-mediated cytokine production, expression levels of IL-2, IL-4, IL-10, IL-12, and IFN-γ in spleen were analyzed using ELISA kits.

**Figure 4:**
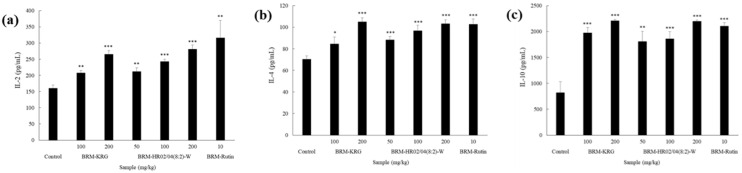

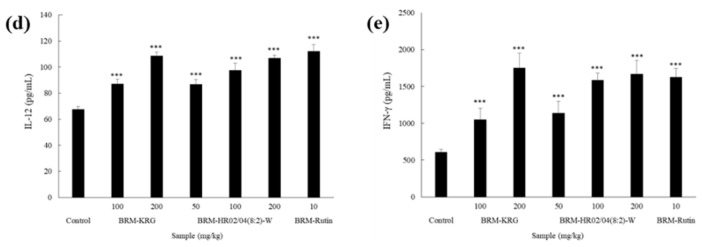
Effects of HR02/04(8:2)-W on IL-2 (**a**), IL-4 (**b**), IL-10 (**c**), IL-12 (**d**), and IFN-γ (**e**) production in spleen. After oral administration for 2 weeks, production of IL-2, IL-4, IL-10, IL-12, and IFN-γ were measured in the medium of splenocytes incubated in 96-well plates for 48 h. Data are shown as mean ± SD. * *p* < 0.05, ** *p* < 0.01, and *** *p* < 0.001 compared with the normal control group.

IL-2 is a cytokine that mediates and regulates adaptive immunity. It is secreted during T lymphocyte activation, which has been reported to stimulate the proliferation and differentiation of T lymphocytes, B lymphocytes, and NK cells [37]. Furthermore, IL-6 has been shown to play a significant role in stimulation of the innate and acquired immune responses by mediating short-range interaction between target cells and T cells, thereby contributing to activation of B lymphocytes and promotion of IgE production [38]. Similarly, IL-10 is a cytokine that regulates innate as well as acquired immunity and has been reported to be expressed by activated macrophages and T lymphocytes. Prior studies have suggested the possibility of a direct role of IL-10 in growth, differentiation, activation, and function of T cells and thymocytes [39,40]. IL-12 mediates innate immunity by promoting Th1 cells and plays an important role in initiating the immune response of macrophages, NK cells, and T lymphocytes. Studies suggest that IL-12 is usually produced in macrophages and dendritic cells [41]. On the other hand, IFN-γ is one of the lymphokines that is involved in the generation of Tc cells from resting precursor cells. IFN-γ has been shown to be produced by activated T cells and NK cells. Additionally, prior studies have suggested that IFN-γ enhances the release of IL-1, a lymphokine that acts as an accessory signal in T cell activation [42,43]. As shown in Figure 4, all types of evaluated cytokines were significantly upregulated on treatment with HR02/04(8:2)-W (*p* < 0.05). Moreover, since expression of all cytokines, except IL-10, was observed to elevate to a level equal to or higher than that expressed by the KRG-treated group, it was believed that the immune response could be effectively mediated.

### 3.6. Effect of HR02/04(8:2)-W on NO Production in Splenocytes

**Figure 5:**
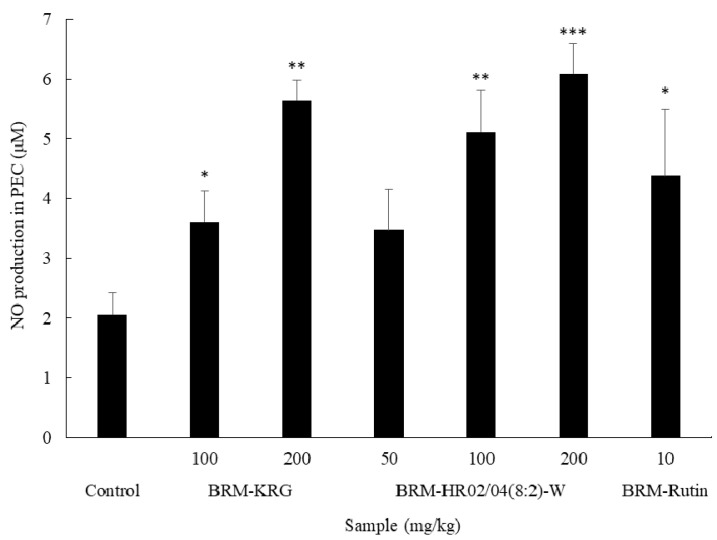
Effect of HR02/04(8:2)-W on NO production in PEC. After oral administration for 2 weeks, peritoneal exudate cell (PEC) was incubated for 2 h to attach macrophages and washed three times with HBSS solution. After 72 h of incubation, NO in the culture medium was assayed by the Griess method. Data are shown as mean ± SD. * *p* < 0.05, ** *p* < 0.01, and *** *p* < 0.001 compared with the normal control group.

The results of measuring the NO production in PEC are shown in Figure 5. The HR02/04(8:2)-W treated groups produced significantly higher amounts of NO than the control group (*p* < 0.05). Furthermore, at a concentration of 100 mg/kg, the KRG treated group showed NO production of 3.6 μM and the HR02/04(8:2)-W treated group showed 5.12 μM, which increased by 42% or more than the KRG treated group. As a result of the experiment, it was confirmed that HR02/04(8:2)-W is a material that can effectively increase the immune response while increasing the amount of NO produced, similar to the result of measuring NO production in RAW264.7 cells.

### 3.7. Effect of HR02/04(8:2)-W on Total Cell Number 

**Figure 6:**
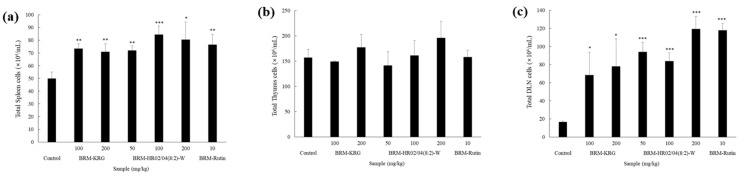

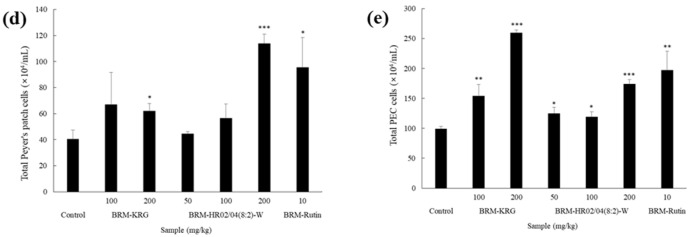
Effects of HR02/04(8:2)-W on the number of total cells in the spleen (**a**), thymus (**b**), DLN (**c**), peyer’s patch (**d**), and PEC (**e**). After oral administration for 2 weeks, absolute cell numbers of spleen, thymus, DLN, peyer’s patch, and PEC were counted manually using a hemocytometer chamber. Data are shown as mean ± SD. * *p* < 0.05, ** *p* < 0.01, and *** *p* < 0.001 compared with the normal control group.

To evaluate the effect of HR02/04(8:2)-W on the immune system, we extracted tissues from major immune organs of mice (spleen, thymus, DLN, Peyer’s patches, and PEC) and measured the total cell number. The spleen is responsible for the key protective immune response against antigens and is known to be the main lymphoid organ where B and T lymphocytes differentiate. Thus, the growth of the spleen cells is considered to be of utmost significance in the immune system [44]. The thymus is one of the central lymphoid organs and has been shown to play an important role in cellular immunity by producing mature T lymphocytes from immature T lymphocytes [45]. Furthermore, Peyer’s patches are considered to be a lymphoid tissue, which induces mucosal immune responses such as local IgA production and systemic immune responses [46]. Lymph nodes are situated partially along the lymphatic vessels and act as a filter station to prevent transmission of pathogens into the body. These have been shown to be the main sites of the innate immune response, which later also mediate an adaptive immune response [47]. Our data analysis showed that compared to the control group, the total number of cells in the HR02/04(8:2)-W-treated group was significantly augmented in all tissues except the thymus (Figure 6). On comparing the effect of KRG and HR02/04(8:2)-W treatment in DLN at concentrations of 100 mg/kg and 200 mg/kg, we found that the KRG-treated group had 68.6 × 10^4^ cells/mL and 78.0 × 10^4^ cells/mL, respectively, while the HR02/04(8:2)-W-treated group had 84.0 × 10^4^ cells/mL and 119.5 × 10^4^ cells/mL, respectively. Thus, it was confirmed that the HR02/04(8:2)-W-treated group significantly increased the number of DLN cells compared to those in the KRG-treated group. In Peyer’s patches, the 200 mg/kg KRG-treated group was found to have 62.0 × 10^4^ cells/mL and the HR02/04(8:2)-W-treated group at the same concentration was observed to have 114.0 × 10^4^ cells/mL. Thus, results suggested that HR02/04(8:2)-W treatment significantly and effectively increased the total cell number in Peyer’s patches by more than 83% compared to that in the KRG-treated group.

### 3.8. Effect of HR02/04(8:2)-W on Albsolute Nunber of Immune Cell Subtypes

#### 3.8.1. Immune Cell Activity and Number in the Spleen

Expression of immune cell subtypes in the spleen tissue was analyzed by flow cytometry (Table 2). The number of CD3+ T cells, CD49b+ NK cells, CD4+ Th cells, and CD8+ T cells in the spleen showed a tendency to increase compared to that in the control group. CD4+/CD25+ and CD8+/CD25+ are known as specific markers for regulatory T (Treg) cells and are stimulated by cytokines such as IL-4 and IL-10. Moreover, they are known to suppress excessive inflammatory reactions by regulating immune responses [48]. In our study, the HR02/04(8:2)-W-treated group showed a tendency to significantly enhance the expression level of CD4+/CD25+ Treg cells compared to that in the control group. This indicated that HR02/04(8:2)-W could effectively regulate overall immune function not only by increasing the immune response in the body, but also by preventing the generation of an excessive immune response. Furthermore, CD23 has been shown to be expressed by mature B cells, activated macrophages, and eosinophils and thereby regulate IgE synthesis [49]. Studies have shown that B220, an isoform of CD45, is not expressed in humans, but is a B cell marker in mice. Furthermore, B lymphocytes are known to be involved in humoral immunity by producing IgE antibodies [50]. On evaluating CD23+/B220+ B cells, we found that the HR02/04(8:2)-W-treated group at all concentrations showed significantly higher expression levels compared to the control group. This suggested that HR02/04(8:2)-W not only stimulated a cell-mediated immune response, but also stimulated a humoral immune response, which could assist in enhancing the immune function. Gr-1+/CD11b+ myeloid-derived suppressor cells are known as bone marrow-derived granulocytes that can differentiate into mature macrophages [51]. On administration of HR02/04(8:2)-W, Gr-1+/CD11b+ expression was found to be significantly upregulated in the HR02/04(8:2)-W-treated groups at all concentrations compared to that in the control group.

**Figure 7:**
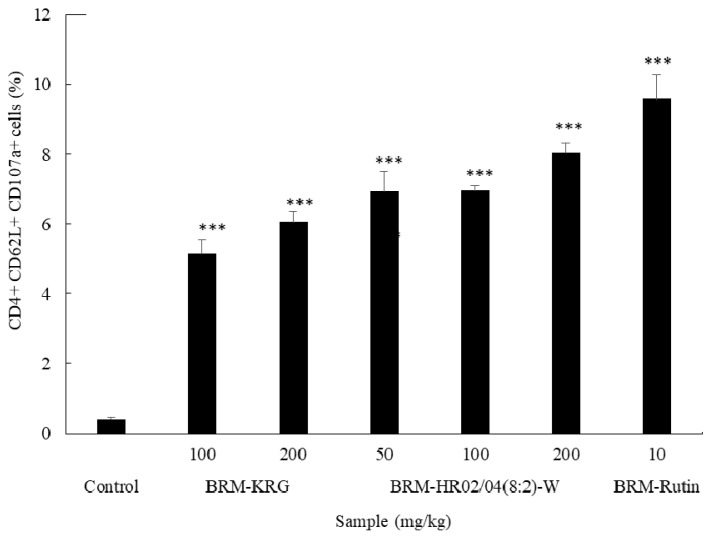
Effect of HR02/04(8:2)-W on NK cell functional activity. Data are shown as mean ± SD. *** *p* < 0.001 compared with the normal control group.

Functional activity of NK cells using CD107a (LAMP-1) as a marker is shown in Figure 7. CD62L is a cell surface marker that has been shown to be expressed by NK cells since its origination and has been found to gradually increase its expression in a time-dependent manner [52]. CD107a (LAMP-1) has been reported as a marker for degranulation of NK and activated CD8+ T cells [53]. CD4+/CD62L+/CD107a+ NK cells were analyzed to investigate the activity of NK cells in the spleen. Our analysis showed that CD107a expression level in NK cells was significantly weak in the control group; however, we found a prompt increase following HR02/04(8:2)-W treatment. Moreover, the level of NK cell activation was found to be significant in the HR02/04(8:2)-W-treated group compared to that in the KRG-treated group (positive control). 

#### 3.8.2. Immune Cell Activity and Number in DLN

Expression analysis of immune cell subtypes in DLN is shown in Table 3. Expression levels of CD3+ T cells, CD49b+ NK cells, CD4+ Th cells, and CD8+ T cells in DLN were found to be significantly upregulated in the HR02/04(8:2)-W-treated group compared to those in the control group. Specifically, on HR02/04(8:2)-W treatment, expression of CD4+/CD25+ Treg cells and CD23+/B220+ B cells was observed to be significantly augmented compared to that in the control group, which was consistent with the experimental results observed in the spleen. 

#### 3.8.3. Immune Cell Activity and Number in PEC, Thymus, and Peyer’s Patches

Expression analyses of immune cell subtypes in PEC, thymus, and Peyer’s patches are shown in Table 4. In PEC, CD3+/CD4+ Th cells have been shown to activate monocytes, macrophagocytes, and NK cells by self-secreted cytokines and thereby function as tumor-killer T cells via an immune response. Additionally, they have been reported to secrete IL-2 to activate CD3+/CD8+ Tc lymphocytes and subsequently support the tumor immune response [54]. Our study confirmed that the number of CD3+/CD4+ Th cells was significantly upregulated in the HR02/04(8:2)-W-treated group compared to that in the control group at a concentration of 50 mg/kg and 100 mg/kg. Moreover, the number of CD3+/CD8+ Tc lymphocytes was found to upsurge with an increase in CD3+/CD4+ Th cells. Further, we observed that CD4+/CD25+ Treg cells decreased significantly in PEC as opposed to its concentration in the spleen. When CD8+/CD25+ Treg cells were treated with 50 mg/kg and 100 mg/kg of HR02/04(8:2)-W, we did not find any significant difference. However, it was confirmed that the number of CD8+/CD25+ Treg cells increased following treatment with a high concentration (200 mg/kg) of HR02/04(8:2)-W. Further, B220+/CD23+ B cells were found to significantly upregulate following HR02/04(8:2)-W administration at any concentration compared to those in the control group. CD69 has been reported as a typical early marker for lymphocyte activation due to its prompt appearance on the surface after stimulation [55]. Furthermore, CD11b, also called MAC-1, along with CD18, is known as a representative surface marker for macrophages [56]. On analyzing the absolute cell number of activated B cells and macrophages by evaluating B220+/CD69+ and CD11b+/CD69+ expression, B220+/CD69+ cells were found to be significantly increased in the HR02/04(8:2)-W-treated group at all concentrations compared to the control group. In the case of CD11b+/CD69+ cells, 50 mg/kg and 100 mg/kg concentrations of HR02/04(8:2)-W did not show a significant difference compared to the control group; however, cell numbers were observed to increase significantly at a concentration of 200 mg/kg. On evaluating the absolute cell number of CD4+ Th cells in thymus based on HR02/04(8:2)-W administration, no significant difference was found at low concentrations of 50 mg/kg and 100 mg/kg compared to the control group. However, a significant effect was observed on administration of 200 mg/kg HR02/04(8:2)-W. In Peyer’s patches, although we were unable to confirm the significant difference of absolute cell number of CD3+ T cells between the control group and KRG-treated group, there was a significant increase in CD3+ T cell number at all concentrations in the HR02/04(8:2)-W-administered group compared to the control group. Both CD3+/CD4+ Th cells and CD3+/CD8+ Tc cells did not show any significant difference from the control group at low HR02/04(8:2)-W concentrations of 50 mg/kg and 100 mg/kg. However, on treating with 200 mg/kg HR02/04(8:2)-W, there was a significant increase in CD3+/CD4+ Th cells and CD3+/CD8+ Tc cells, which was observed to be more than three-fold. From these results, we confirmed that HR02/04(8:2)-W can improve immune function by effectively regulating the expression of cells involved in innate and adaptive immune responses in PEC, thymus, and peyer’s patch.

## 4. Conclusions

The objective of this study was to develop a mixture of natural products that could significantly improve immune function and to evaluate the efficacy of different combinations. We used FES and SQN to prepare samples of different mixing ratios (10:0, 2:8, 5:5, 8:2, and 0:10) and extraction conditions (ethanol extraction and hot water extraction). By evaluating the expression level of NO and cytokine production in RAW264.7 cells, one of the mixing ratios [HR02/04(8:2)-W] with significant efficacy to enhance immunity in vitro was selected. To confirm the immune-enhancing efficacy of HR02/04(8:2)-W, immunity-related biomarkers were assessed in BALB/c mice after oral administration of the concoction for two weeks. We found that administration of HR02/04(8:2)-W and its indicator component, rutin, effectively augmented the absolute cell count and cytotoxicity of BALB/c mice-derived NK cells. On evaluating key cytokines (IL-2, IL-4, IL-10, IL-12, and IFN-γ) related to the immune response in cultured spleen cells, we found a significant increase in the expression levels of cytokines in the HR02/04(8:2)-W-administered group at all concentrations and the rutin-administered group, which were similar to that of the KRG-treated group (positive control). On analyzing NO production, which is the main metabolite of the immune response in mouse spleen cells, we found a remarkable and effective increase in NO concentration, with a significant difference from the control group. These results are similar to those of in vitro experiments using RAW264.7 cells, and it was confirmed that HR02/04(8:2)-W effectively increases NO production and stimulates immune activity. Furthermore, on evaluation of total number of cells in major immune response-related tissues of the body (spleen, thymus, DLN, Peyer’s patches, and PEC), we found that DLN, spleen, and PEC had significantly increased the number of cells in all HR02/04(8:2)-W-administered groups compared to those in the control group. In Peyer’s patches, a significant increase in cell number was not observed at low concentrations of 50 mg/kg and 100 mg/kg HR02/04(8:2)-W. However, a more than two-fold increase in cell number was observed at a concentration of 200 mg/kg HR02/04(8:2)-W. Conversely, the total number of cells in thymus did not prove to be significantly different in positive control (KRG) and HR02/04(8:2)-W treatments. On further analysis of the immune cell phenotype in major immune tissues using FACS, absolute cell numbers of lymphocytes such as Th cells, Tc cells, Treg cells, B cells, and macrophages were found to be effectively regulated following HR02/04(8:2)-W treatment. In particular, it was confirmed that the expression of B lymphocytes was effectively increased in all tissues. When the results of HR02/04(8:2)-W treatment increased in IL-6 production in the in vitro experiment of this study, this increase in B lymphocytes is thought to be due to an increase in IL-6, which differentiates B lymphocytes to induce antibody production. Thus, our results confirmed the effect of immunity enhancement on the administration of the prepared mixture [HR02/04(8:2)-W]. Overall, we believe that HR02/04(8:2)-W has the potential to be a reliable natural resource that can assist in improving the immune function of the body.

## Figures and Tables

**Table 1:** The samples used in the experiment and extraction yield.

Name	Mixture Ratio FES ^(1)^:SQN ^(2)^	Extraction Solvent	Extraction Yield (%)
HR1902-W	10:0	Water	27.0
HR1902-70E		70% Ethanol	22.1
HR02/04(2:8)-W	2:8	Water	24.3
HR02/04(2:8)-70E		70% Ethanol	17.3
HR02/04(5:5)-W	5:5	Water	16.9
HR02/04(5:5)-70E		70% Ethanol	21.8
HR02/04(8:2)-W	8:2	Water	16.2
HR02/04(8:2)-70E		70% Ethanol	19.3
HR1904-W	0:10	Water	23.1
HR1904-70E		70% Ethanol	16.0

^(1)^ FES: Ficus erecta var. sieboldii.; ^(2)^ SQN: Sasa quelpaertensis Nakai.

**Table 2:** Quantification by means of FACS analysis of various immune cell subtypes in the spleen.

Spleen (×10^5^ Cells)	Immune Cell Number in BALB/c Mice (Absolute No.)						
	BALB/c	BRM-KRG		BRM-HR02/04(8:2)-W			BRM-Rutin
	Normal	100 mg/kg	200 mg/kg	50 mg/kg	100 mg/kg	200 mg/kg	10 mg/kg
CD3^+^	25.5 ± 1.40	40.7 ± 1.56 **	41.9 ± 3.66 **	45.1 ± 1.40 **	57.2 ± 7.70 **	55.5 ± 8.72 **	49.0 ± 2.78 ***
CD49b^+^	1.7 ± 0.04	2.5 ± 0.11 ***	2.4 ± 0.12 **	2.0 ± 0.14	2.8 ± 0.28 **	2.7 ± 0.49	3.1 ± 0.09 ***
CD4^+^	9.8 ± 1.42	16.4 ± 0.66 ***	17.1 ± 2.07 **	18.2 ± 1.65 **	19.4 ± 1.25 ***	17.7 ± 2.63 **	17.7 ± 1.19 ***
CD8^+^	6.4 ± 0.30	11.2 ± 0.83 ***	13.0 ± 1.07 ***	11.0 ± 0.64 ***	9.8 ± 0.45 ***	9.3 ± 1.80	9.1 ± 0.06 ***
CD4^+^/CD25^+^	1.2 ± 0.24	2.1 ± 0.18 **	2.2 ± 0.40 *	2.6 ± 0.18 ***	2.2 ± 0.17 **	2.4 ± 0.67	2.0 ± 0.33
CD8^+^/CD25^+^	0.4 ± 0.02	0.4 ± 0.01 **	0.8 ± 0.03 ***	0.5 ± 0.11	0.4 ± 0.07	0.5 ± 0.13	0.5 ± 0.10
CD23^+^/B220^+^	12.5 ± 1.23	20.7 ± 1.25 ***	19.4 ± 2.13 **	18.8 ± 0.44 ***	25.2 ± 1.44 ***	25.9 ± 4.17 **	21.7 ± 2.73 **
Gr-1^+^/CD11b^+^	1.3 ± 0.42	3.8 ± 0.53 **	4.1 ± 0.13 ***	2.9 ± 0.05 **	3.5 ± 0.07 ***	3.6 ± 0.71 **	5.1 ± 0.21 ***

Results are expressed as mean ± S.E. Statistical significance between the control and treatment groups was assessed by student *t*-test (* *p* < 0.05, ** *p* < 0.01 and *** *p* < 0.001).

**Table 3:** Quantification by means of FACS analysis of various immune cell subtypes in draining lymph node (DLN).

DLN (×10^5^ Cells)	Immune Cell Number in BALB/c Mice (Absolute No.)						
	BALB/c	BRM-KRG		BRM-HR02/04(8:2)-W			BRM-Rutin
	Normal	100 mg/kg	200 mg/kg	50 mg/kg	100 mg/kg	200 mg/kg	10 mg/kg
CD3^+^	9.3 ± 2.07	55.8 ± 19.97 *	67.2 ± 27.19 *	76.8 ± 9.67 ***	70.0 ± 7.08 ***	93.7 ± 30.27 **	95.9 ± 2.35 ***
CD49b^+^	0.3 ± 0.00	1.0 ± 0.38	0.6 ± 0.15	0.8 ± 0.05 ***	0.9 ± 0.03 ***	1.0 ± 0.25 **	1.1 ± 0.29 **
CD4^+^	7.3 ± 0.70	38.6 ± 13.64 *	44.5 ± 18.34 *	53.4 ± 6.19 ***	45.7 ± 9.00 ***	63.1 ± 20.29 **	63.8 ± 1.60 ***
CD8^+^	3.6 ± 0.61	16.7 ± 5.94 *	21.0 ± 8.67 *	21.3 ± 2.76 ***	19.6 ± 3.89 ***	26.0 ± 8.73 **	27.3 ± 0.27 ***
CD4^+^/CD25^+^	1.1 ± 0.22	6.5 ± 2.22 **	8.3 ± 3.71	8.6 ± 1.07 ***	8.9 ± 1.54 ***	11.7 ± 4.15 **	10.4 ± 0.30 ***
CD23^+^/B220^+^	0.7 ± 0.13	4.3 ± 1.30 **	12.1 ± 5.62 *	6.3 ± 0.61 ***	6.9 ± 0.96 ***	7.8 ± 1.93 *	7.3 ± 1.35 ***

Results are expressed as mean ± S.E. Statistical significance between the control and treatment groups was assessed by student *t*-test (* *p* < 0.05, ** *p* < 0.01 and *** *p* < 0.001).

**Table 4:** Quantification by means of FACS analysis of various immune cell subtypes in PEC, Thymus, and peyer’s patches.

PEC, Thymus, Peyer’s Patches		Immune Cell Number in BALB/c Mice (Absolute No.)						
		BALB/c	BRM-KRG		BRM-HR02/04(8:2)-W			BRM-Rutin
		Normal	100 mg/kg	200 mg/kg	50 mg/kg	100 mg/kg	200 mg/kg	10 mg/kg
CD3^+^/CD4^+^	PEC (×10^5^ cells)	7.9 ± 0.20	28.9 ± 10.66	35.3 ± 0.63 ***	12.0 ± 1.17 **	11.8 ± 2.68	29.7 ± 1.24 ***	32.8 ± 6.94 **
CD3^+^/CD8^+^		5.3 ± 0.10	10.5 ± 1.99 **	16.0 ± 0.41 ***	5.9 ± 0.07 ***	5.2 ± 0.80	12.6 ± 0.58 ***	11.8 ± 1.73 **
CD4^+^/CD25^+^		3.1 ± 0.04	5.1 ± 0.98 *	3.6 ± 0.86	2.4 ± 0.43	2.3 ± 0.22 **	4.2 ± 0.74	7.3 ± 1.12 **
CD8^+^/CD25^+^		0.5 ± 0.01	1.0 ± 0.12 ***	2.1 ± 0.05 ***	0.7 ± 0.16	0.8 ± 0.22	1.3 ± 0.08 ***	1.3 ± 0.31 **
B220^+^/CD23^+^		7.3 ± 0.82	16.1 ± 1.68 ***	33.2 ± 3.54 ***	13.6 ± 1.78 **	18.2 ± 3.09 **	28.5 ± 0.42 ***	27.2 ± 3.73 ***
B220^+^/CD69^+^		7.9 ± 0.14	14.9 ± 1.69 ***	25.2 ± 0.76 ***	12.2 ± 0.49 ***	12.3 ± 0.46 ***	21.6 ± 0.57 ***	41.6 ± 6.55 ***
CD11b^+^/CD69^+^		7.9 ± 0.32	10.2 ± 2.76	20.3 ± 4.20 **	8.9 ± 0.82	6.9 ± 1.12	11.8 ± 0.04 ***	21.5 ± 4.46 **
CD4^+^	Thymus (×10^6^ cells)	9.8 ± 1.00	12.8 ± 0.38 **	16.1 ± 2.96 **	11.2 ± 3.00	9.3 ± 0.59	18.6 ± 2.40 **	12.6 ± 1.82
CD3^+^	Peyer’s patch (×10^5^ cells)	7.4 ± 1.28	10.0 ± 5.91	11.4 ± 3.74	10.4 ± 0.81 *	11.7 ± 1.38 *	37.3 ± 3.76 ***	26.8 ± 5.02 **
CD3^+^/CD4^+^		2.2 ± 0.67	2.8 ± 1.40	2.8 ± 0.94	3.0 ± 0.35	3.7 ± 0.62	7.1 ± 1.01 **	4.1 ± 0.64 *
CD3^+^/CD8^+^		2.5 ± 0.32	2.8 ± 1.18	4.1 ± 1.05	3.8 ± 0.82	4.9 ± 1.96	9.8 ± 2.04 **	11.0 ± 3.25 **

Results are expressed as mean ± S.E. Statistical significance between the control and treatment groups was assessed by student *t*-test (* *p* < 0.05, ** *p* < 0.01 and *** *p* < 0.001).
